# The microbial *terroir*: open questions on the Nagoya protocol applied to microbial resources

**DOI:** 10.1111/1751-7915.13839

**Published:** 2021-07-26

**Authors:** Javier Pascual, Kristie Tanner, Cristina Vilanova, Manuel Porcar, Ana Delgado

**Affiliations:** ^1^ Darwin Bioprospecting Excellence S.L. Paterna Spain; ^2^ Institute for Integrative Systems Biology UV‐CSIC Paterna Spain; ^3^ TIK Centre for Technology, Innovation and Culture University of Oslo Oslo Norway

## Abstract

The Nagoya Protocol on Access and Benefit‐sharing (https://www.cbd.int/abs/), primarily designed for vascular plant and animal resources, is also extended to the use of microbial resources, but its application to the microbiological realm has raised many doubts and provoked criticisms. This is because of the particularities of microbial ecology and the technical and legal difficulties encompassed in its application.

## Introduction

*Terroir *(/tɛˈrwɑːr/, French: [tɛʁwaʁ]; from terre, ‘land’) is a French term widely used to describe the factors in the environment that affect the phenotype of a crop (typically, a vineyard), including the geographic context and farming practices. A Bordeaux or a Rioja are examples of wines that are named, labelled and commercialized based on their geographical origin. The concept of *terroir* is often linked to the (belief of) existence of specific quality features in a given product than can only be found in a specific geographic location, because they are inherently linked to it. We use this metaphor of ‘microbial terroir’ as a way to introduce some key challenges regarding the regulation of the isolation and use of microorganisms under the Convention on Biological Diversity‐Nagoya Protocol.

Questions are many and opened, both for scientists, companies and policymakers. The ambition of this paper was not to answer those questions but to provide a quick picture (a map) of microbial controversies that we have identified over the years in the literature and conversations with concerned colleagues who, often, do not know to which extent the Nagoya Protocol applies to their research. We aim here at formulating these controversies in a way that is accessible to practitioners in microbiology as well as to those working in policy and the law. We are convinced that part of the problem relies on the lack of a hybrid language, one that enables us to talk about the biological, social and technical complexities. *Terroir* may be a good place to start, as it refers to complex biological and social–technical complexities, which is precisely what is shaping the controversies that we briefly map in what follows:

## Microorganisms as *in situ* biodiversity? Where are they from?

The CBD‐Nagoya Protocol covers access and use of genetic resources, and it presumes that genetic material results of the adaptation to specific geographical sites within particular national jurisdictions. However, the biogeography of macroorganisms differs greatly from that of microorganisms: while the former can be found forming local endemisms and biodiversity hotspots, the latter tend to be more ‘cosmopolitan’. In other words, large animals and plants tend to inhabit specific locations on Earth, while microorganisms appear as vastly geography‐independent. This leads to the question of whether the Nagoya protocol is suited to cover the microbial realm (Overmann and Scholz, 2017).

The cosmopolitan nature of microorganisms (also known as the global microbial connectivity issue) is mainly due to the ease with which they are transported, for example, via animals, dust particles, aquatic systems or air streams, in such a way that very similar genotypes can be found in locations thousands of kilometres away. This is the case, for example, of two *Phaeobacter gallaciensis* strains isolated from the Atlantic coast of Spain and from marine waters near Sydney that displayed up to 93% shared genes (Thole *et al*., 2012). More interestingly, microorganisms isolated from different regions can be producers of the same biomolecules. This is the case of the xantholysin A, a cyclic lipopeptide with antimicrobial and antitumor activity produces by *Pseudomonas* strains phylogenetically related isolated from both Sri Lanka (Li *et al*., 2013) and Europe (Pascual *et al*., 2014).

Both countries in the Global North and South are providers of microbial biodiversity (Overmann and Scholz, 2017), while mainly Global South countries are identified as biodiversity hotspots for plant and animal varieties. However, it has to be stressed that, unlike large plants and animals, microorganisms live in microniches, which may be very similar in different regions of the world. This is the case of the microbial communities inhabiting solar panel surfaces, which revealed to be very similar despite the large distance between sampling sites (Porcar *et al*., 2018). Additionally, bacterial genomes are not stable, since the exchange of biosynthetic gene clusters via horizontal transfer is frequent among microorganisms.

All this leads to intriguing questions: What if two bacterial strains are isolated in two distant countries but prove to be genetically identical or are able to produce the same biomolecule with biotechnological applications? What if one of these countries regulates access to its genetic resources through the Nagoya protocol but the other does not?

## Microbial Multi‐Origin Constructs and International Cooperation in Research

And what about GMOs or Synthetic Biology‐issued (SynBio) agents? If the Nagoya protocol raises many concerns on the exploitation of natural microbial strains, its application to genetically engineered microorganisms is even more complicated. Unlike traditional GMOs, in which commonly single genes are introduced in a host species, SynBio agents are built by using a combination of many parts of different origins and, therefore, with potentially different ownership claims (Manheim, 2016; Scott and Berry, 2018). Up to date, the traditional bacterial chassis for genetic modifications has been *Escherichia coli*, but, considering the massive bacterial biodiversity, a large number of bacterial species may be much better suited than *E. coli* and it is very likely that new bacterial species will arise as agents to express SynBio constructs in several industrial or environmental contexts. In a scenario of complex biological parts and chassis, a question that arises is the identification of the country/countries that should be considered as the origin(s) of resulting engineered microorganism. If a Synbio agent is made using a chassis from a given country and modified with exogenous DNA parts originating from other regions of the world, which is the geographic location that should apply to the final construct? And, if all the countries involved are considered, which criteria should be used to weight their relative contribution? In this sense, it has to be stressed that an imbalance between function and genomic weight will very likely exist, in the sense that minor sequences (in terms of number of nucleotidic bases) can result in a major behavioural change (in the sense of biotechnological output ‐and benefit). Therefore, is it the ‘amount’ of exogenous DNA or the ‘importance’ of its function what should be considered in the frame of the Nagoya Protocol? If the latter applies, the different nature of the DNA parts composing the constructs (not only coding sequences, but also non‐coding regions that are essential to regulate gene expression) adds even more complexity to define such ‘relative weight’ in benefit sharing (Bagley and Rai, 2013).

On the other hand, the development of complex genetic circuits, multi‐device systems and microbial chasses is a task that requires international cooperation, and in fact sharing standardized elements is central to most SynBio projects. Therefore, is it fair that benefit sharing is only applied to the country where the genetic resources originated, even though the resulting standard parts are achieved with coordinated efforts (including economic investments) from other countries?

## Microbial collections: Digital data and the pressure towards open science

Advances in gene‐sequencing technology and cultivation‐free screening of microbial strains have greatly augmented the prospects and promise of microbial bioprospecting on a planetary scale. As sequencing technology rapidly develops, sequence data have proliferated to a great degree, and most microbial collections exist today as digital collections. Much of this sequence information is published on public databases, and this adds an ultimate layer of complexity to the debate on the Nagoya Protocol and microorganisms: what happens if we consider that microorganisms, or at least their genomic information, also *exist* in online databases? An up‐to‐date example on this is the COVID vaccine: SARS‐CoV‐2 was first sequenced in China. Publicly available, this sequence information has enabled researchers in other countries to start designing vaccines for profit. Open data can be accessed from any location, which also opens questions in the context of the Nagoya Protocol about how to regulate access and use. Questions about how much ‘subsidiary’ information (including ecological and geographical data) should be tagged to digital data, how to improve metadata annotation or the need to enhance sequence data tracing technologies remain open (CBD‐Ad hoc, 2018, 2020; Ambler *et al*., 2021). The possibility of tracing data from a database to the original source is key for the implementation of the Nagoya Protocol, but this presents already technical challenges for microorganisms. If microbial strains can be found in different locations, one could identify an interesting sequence in a location and then collect the actual material counterpart in a country with relaxed regulations on access and use (Delgado, 2021). To sum up with a final, open question: in a world with a nearly ubiquitous distribution of natural microbial resources, with complex engineered strains made with parts of multiple origin, and with genetic information having the cloud as the closest geographical location, is there any room for the concept of *terroir* when it comes to microorganisms?

## Conflict of interest

None declared.
